# Therapeutic controversies over use of antioxidant supplements during cancer treatment: a scoping review

**DOI:** 10.3389/fnut.2024.1480780

**Published:** 2024-12-09

**Authors:** Mulugeta Woldeselassie, Aynadis Tamene

**Affiliations:** ^1^College of Medicine and Health Sciences, Wollo University, Dessie, Ethiopia; ^2^Food Science and Nutrition, Addis Ababa University, Addis Ababa, Ethiopia

**Keywords:** antioxidant, cancer, therapeutic controversy, treatment, supplements, use

## Abstract

**Background:**

Antioxidant supplements are widely used during cancer treatment to prevent oxidative stress, reduce treatment toxicities, and improve patient outcomes. However, current literature reveals significant gaps suggesting that antioxidants may protect both healthy and tumor cells from oxidative damage, thereby reducing treatment efficacy. It is for this reason that antioxidant supplements have become a source of therapeutic controversy.

**Objective:**

To review therapeutic controversies over the use of antioxidant supplements during cancer treatment.

**Methods:**

Scoping review of the international published articles following the Arksey and O’Malley framework, cross-sectional studies, clinical and pre-clinical studies, systematic and umbrella reviews and grey literatures published from 2014 to 2024 with all age patient populations were included. A structured literature search was conducted of CINAHL, EMBASE, MEDLINE, Google Scholar, using key medical subject heading words and Cochrane Collaboration and Joanna Briggs Institute databases. All included studies were reviewed independently by two investigators. Data were extracted, collated by type of antioxidants, summarized in tables and synthesized for analysis.

**Result:**

A total of 1, 550 articles were identified. After reviewing all literatures, twenty-one (21) were full-text articles, grey literatures (2), and systematic reviews (42) and umbrella reviews (3), met the criteria for inclusion. In this review, the use of antioxidant supplements can benefit cancer cells in the same way as they do for normal cells during cancer treatment. In addition, not all antioxidants were effective in inhibiting oxidative stress, reduce treatment toxicities, and improve patient outcomes.

**Conclusion and recommendations:**

According to this review, the use of antioxidant supplements can benefit tumor cells in the same manner as they do for normal cells. Therefore, oncologists should advise not to take antioxidant supplements during chemotherapy and/or radiotherapy. Future research including potential clinical and preclinical trials, mechanistic studies, and exploration of different vitamin and mineral supplement studies are required to uncover the complete potential of antioxidant supplements for cancer treatment or determine their safety and effectiveness when used alongside standard cancer treatments. Furthermore, the results of this review could be used for future systematic review of therapeutic controversies over use of antioxidant supplements during cancer treatment.

## 1 Introduction

Antioxidants are substances that prevent, delay, or remove the oxidative damage caused by reactive oxygen species (ROS) (1). These reactive oxygen species are produced by living cells as a metabolic byproduct and play a significant role as signaling molecules throughout the entire cell death process (2).

An excess of ROS can destroy organelles’ structure and biomolecules, leading to inflammatory responses and mutagenesis, and this is a known mechanism for the development of cancer (2, 3).

The term “cancer” refers “uncontrolled growth of abnormal cells in the body (4). After cardiovascular disease, cancer was the second-leading cause of death worldwide in 2018, with 9.6 million fatalities (5).

Cancer is believed to be the cause of 13.4% of fatalities globally (6).

By 2030, there will be 23.6 million new cancer cases globally each year, which is 68% more cases than present if existing patterns in the prevalence of major cancers and population growth are seen worldwide (7).

However, when cancer has developed, the death of malignant cells by treatment with chemotherapeutic drugs and radiation that produce free radicals in the cells, such as hydrogen peroxide and superoxide, mostly depends on their oxidative damage (8).

Chemotherapy and radiation therapy can cause the production of reactive oxygen species, which can damage DNA or interfere with DNA replication machinery through direct and indirect effects on malignant cells. This can lead to aberrations in various cellular signaling pathways, which can result in cellular toxicity and death from chemotherapy or radiation therapy (9, 10).

This being said, one potential remedy might involve use of antioxidants, which are mostly taken as supplements, together with radiation therapy and/or chemotherapy that causes cellular oxidative damage (11, 12). The primary antioxidants that help in preventing free radicals inside the human body are the vitamins (A, C, D, E, beta-carotene, and coenzyme Q10) and minerals (selenium and zinc) (13) (125). These supplements have the potential to shield normal cells from the oxidative stress secondary to some chemotherapy and radiation treatments. However, the same process may protect tumor cells and possibly minimize the effect of cancer therapies (11).

Despite nearly two decades of research, there is still no consensus on whether antioxidant supplementation is beneficial or harmful during cancer treatment. Limited systematic reviews have highlighted the potential risks associated with antioxidant use, suggesting that they may protect both healthy and tumor cells from oxidative damage, thereby reducing treatment efficacy. A comprehensive review would synthesize findings from various studies, including Systematic reviews, umbrella reviews, randomized controlled trials and observational studies, to provide clearer guidance for clinicians and patients alike (13, 14).

In addition, current literature reveals significant gaps regarding the interaction between antioxidant supplements and cancer therapies. Many systematic reviews have focused primarily on the reduction of side effects rather than on therapeutic efficacy. There is a pressing need to systematically assess how these supplements influence treatment outcomes, including safety, efficacy, and recurrence risks. A detailed review could help identify these gaps and propose directions for future research [Risks and benefits of antioxidant supplement use during cancer treatment (12)].

Furthermore, the landscape of cancer treatment is continuously evolving, with new therapies and regimens emerging regularly. Since previous reviews were published, there have been significant changes in both chemotherapy protocols and the types of antioxidant supplements available. An updated review would reflect these advancements and ensure that healthcare providers are equipped with the latest information (12).

Therefore, the objective of the present review was to systematically map out the body of existing literature on therapeutic controversies over the use of antioxidant supplements during cancer treatment in order to identify type of antioxidant supplements that have been commonly given for patients, treatment outcomes reported, summarize the safety and effectiveness of these supplements during cancer treatment in the various clinical settings and opportunities for further research.

## 2 Methods

The review followed established methodological approaches for scoping reviews using the framework presented by Arskey and O’Malley for the conduct of scoping reviews (15). Scoping reviews aim to identify and describe the breadth of literature on a topic when it is either highly complex, involves a broad array of study designs, or when a comprehensive review is being completed for the first time; all of these factors apply to the present review. Scoping reviews aim to map key concepts in a field of study and the available types of evidence. The review is completed in a way that is systematic, highly rigorous, and transparent in order to minimize bias. Briefly, the sequential stages of the process were: identifying the research question, identifying the relevant literature, selecting the literature, charting the data and, collating, summarizing and reporting results.

### 2.1 Identifying the research questions

This review was guided by the following inter-related queries:

What type of antioxidant supplements have been commonly given for patients during cancer treatment in the various clinical settings?

What treatment outcomes have been reported in the published literature?

Based on the reported outcomes, are antioxidant supplements effective for patients during cancer treatment?

### 2.2 Identifying relevant literature

A two-tiered search strategy was used. First, CINAHL, EMBASE and MEDLINE databases were searched for relevant published articles. Hand-searching of reference lists on key papers and web-based search of the grey literature, such as Google Scholar and professional and government websites were performed with the same terms used for the published articles. Systematic reviews were retrieved from the Cochrane Library and the Joanna Briggs Institute EBP database. Second, references from the retrieved systematic reviews were screened to ensure that all relevant primary studies were included in this scoping review. Articles published in English, between January 2014 and December 2024 were retrieved. This time period was selected to enable identification and inclusion of recent literatures. Search terms and linked terms included: antioxidant OR supplement OR vitamin A OR vitamin C OR vitamin D OR vitamin E OR selenium OR zinc OR coenzyme Q10 OR cancer OR treatment OR therapy OR chemotherapy OR radiotherapy OR immunotherapy OR controversies OR oncology OR patients. This search yielded 1, 158 articles. The second-tier search involved examining the bibliographies of retrieved key articles to identify additional relevant studies and seminal articles from the literature; this process identified an additional 392 articles for a total of 1, 550 articles.

### 2.3 Selecting the literature (inclusion and exclusion criteria)

Articles included in this scoping review met specified inclusion criteria: (i) cross-sectional studies, pre-clinical studies, randomized controlled trial design, systematic and umbrella reviews, grey literatures published from 2014 to 2024 and (ii) all age patient populations.

Articles excluded in this scoping review were publications other than English language and those who were in Beall’s list are excluded from this review.

### 2.4 Charting the data

Following initial screening, two reviewers (MWS, AT) extracted three specific components using a standardized form: (i) key elements of the antioxidant supplements, (ii) the most frequently reported outcome measures used to assess therapeutic controversies, and (iii) data evaluating safety and effectiveness of the supplements.

### 2.5 Collating, summarizing, and reporting the literature

We provided a brief history and rationale for the reviewing the topic, a summary of the antioxidant supplements as well as outcome measures and trend of safety and effectiveness of the antioxidant supplements. For each study, outcomes were summarized and reported as better, equivalent or worse than not taking the supplements. In cases where multiple outcome measures were evaluated within the same category, the outcome was reported using majority rule.

#### 2.5.1 Identification of databases and registers

**Figure 1:**
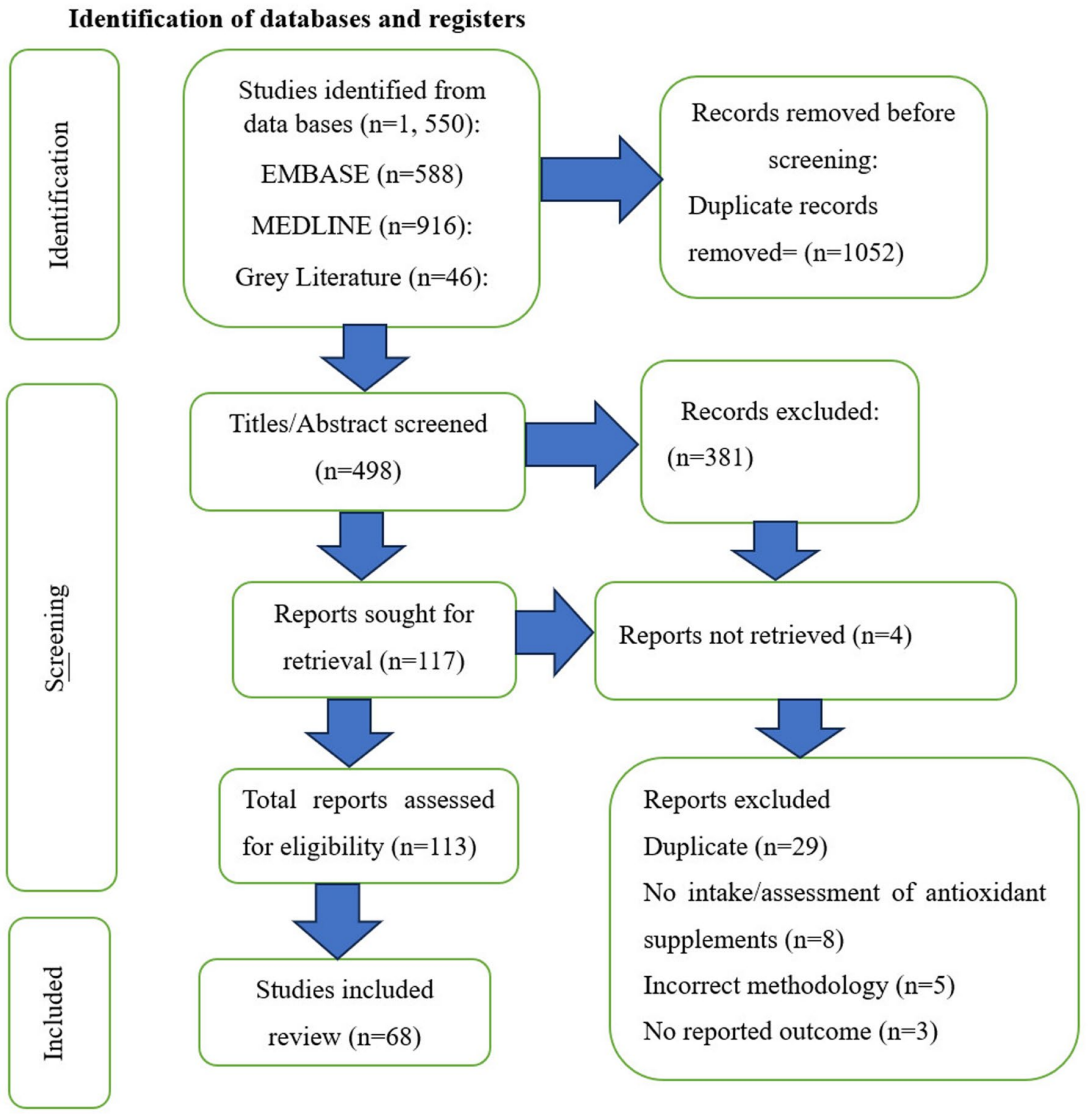
PRISMA flow diagram for the study selection process.

See Figure 1 for PRISMA flow diagram.

## 3 Results and discussion

To the best of our knowledge, this scoping review is the first highlighting the studies that reported the therapeutic controversies of anti-oxidant supplements during cancer treatment. The aim of cancer treatment should be the complete removal of malignant cells and the reduction of therapy-induced cellular toxicity in healthy tissues. Another aim should be the detoxification of detrimental effects after treatment (16). Hence, this review highlights the therapeutic controversies over the use of antioxidant supplements in cancer treatment.

### 3.1 Therapeutic controversies over use of vitamin A supplement during cancer treatment

During cancer treatment, giving patients vitamin A supplements can help improve the overall health and enhance survival rates. It also reduces adverse effects and damage to healthy tissues (17).

In a study with 235 patients with acute promyelocytic disease, they received a combination of arsenic acid and all-trans retinoic acid (45 mg/m^2^). The results showed a lower chance of cancer recurrence and a higher chance of complete cure (18).

A similar study showed that vitamin A supplementation seems safe and does not impede with chemotherapy effectiveness in majority of patients (74).

Another study revealed that giving vitamin A supplementation (8,000 IU/8 h) along with neoadjuvant chemotherapy for advanced cervical carcinoma. This approach showed a significantly improved therapeutic response in these patients (18).

In addition, when treating low-risk gestational trophoblastic neoplasia with methotrexate, oral vitamin A supplements (6,000 IU/day) were found to decrease tumor markers and chemoresistance in these patients (18). Research by Zirpoli et al. (19) found that chemotherapy efficiency was not significantly affected by taking the antioxidant vitamin A.

The EUROSCAN study also reported that retinyl palmitate alone helped prevent primary cancers when given regularly throughout the year. Participants who received vitamin A did not develop secondary cancers during the study period (20).

According to a 2015 review study, there was no evidence to suggest that taking vitamin A supplements alongside cancer therapy is detrimental (13).

On the contrary, meta-analysis research showed that no significant difference in overall survival between individuals who took antioxidant vitamin A during chemotherapy and those who did not (21).

Additionally, taking high doses of vitamin A coupled with beta-carotene daily was associated with an increased risk of lung cancer mortality after 4 years in the CARET trial. There was also an extended risk of death for up to 6 years following supplementation (74).

Moreover, vitamin A supplementation during chemotherapy was linked to a significantly higher risk of death and recurrence in cancer patients (22).

Both radiation and chemotherapy aim to damage malignant cells or increase oxidative stress around them to induce cell death efficiently (23). Meanwhile, antioxidants like vitamin A help repair cell damage by fighting oxidative damage. This function might counteract the expected effects of chemotherapy and radiotherapy by inadvertently protecting cancer cells as well. This discrepancy in goal could significantly increase the risks associated with vitamin A supplement during cancer treatment (75) (Table 1).

**Table 1:** Therapeutic controversies over use of vitamin A supplement during cancer treatment.

Author	Year	Type	Sample size	Review findings
Calvani, Pasha, et al.	2020	Systematic review	189 articles	During chemotherapy, intake of antioxidant supplements potentiates cancer therapy by minimizing detrimental effects, oxidative damage and enhancing patient health.
Talib, Ahmed Jum’AH, et al.	2024	Systematic review	158 articles	A phase 3 clinical trial recruiting patients with acute leukemia showed that intake of all-trans retinoic acid and arsenic acid raised cure and decreased recurrence rates.
Zirpoli, McCann et al.	2017	Prospective cohort	1,225 participants	Intake of vitamin A does not significantly affect the efficacy of chemotherapy.
Meliante, Petrella et al.	2023	Systematic review	65 articles	Vitamin A intake alone adequately inhibits primary malignancy without secondary tumors seen in the intervention group, in spite of having two incidences of secondary cancers in the control group.
Yasueda, Urushima et al.	2016	Systematic review	399 articles	There was no evidence that revealed taking antioxidant supplements together with cancer therapy was harmful.
Andrea S. Blevins Primeau	2017	Systematic review	23 articles	Daily intake of vitamin A and beta-carotene raised the risk of incidence of lung cancer and death after 4 years.
Li, Lin, et al.	2021	Systematic review and meta-analysis	8 studies	During chemotherapy, no significant variations seen between intake of vitamin A and overall survival.
Story, Sabin et al.	2019	Guest post	1 reviewed article	During chemotherapy. Vitamin A intake was linked to a higher risk of death and recurrence.

### 3.2 Therapeutic controversies over use of beta-carotene supplement during cancer treatment

Certain studies showed that ß-carotene can provide protection to the body. It achieves this by boosting the immune system through the activation of T-and B-cells, increasing the number of T-helper cells, and enhancing the activity of natural killer cells (24).

In the context of prostate cancer metastases or death, the consumption of beta-carotene during radiation therapy does not appear to increase the risk. Over a period of 10 years, the group that received the placebo had an 89% survival rate without the disease, while the group that received beta-carotene had a 92% survival rate (25).

A randomized controlled trial involving 264 patients on cancer treatment were assigned into a placebo, while the other group took 50 mg/day of beta-carotene. The investigators followed the patients for 51 months. Finally, no observed differences were seen between the two groups (20).

In addition, similar study also examined 214 patients who took 75 mg/day of beta-carotene regularly for 3 years. After a follow-up period of 59 months, they observed no significant variations in disease-free survival rates or the development of secondary neoplasms (20).

Contrary to this, giving carotenoids with chemotherapy treatment was linked to a higher chance of death and recurrence (22). Similarly, it was also reported that individuals who took carotenoids while undergoing chemotherapy had an increased chance of breast cancer recurrence (26).

Taking antioxidant supplements while undergoing chemotherapy and radiation therapy could potentially result in increased mortality rates and reduced likelihood of remaining cancer-free in breast cancer survivors (27).

In one trial, researchers combined interferon-α with additional medications to treat advanced head and neck cancer that had spread to nearby tissues. The likelihood of developing a second primary tumor was found to be low, but there was a higher occurrence of lung cancers in the experimental group (20).

A long-term clinical trial, spanning 8 years and involving nearly twenty-nine thousand male smokers, aimed to investigate the potential anti-cancer effects of beta-carotene. Astonishingly, male smokers who consumed beta-carotene experienced a 20% higher risk of developing lung cancer (28).

The American Cancer Society states that beta-carotene intake can enhance the strength of white blood cells in our immune system by preventing the harmful effects of free radicals that can harm cells (29).

Radiation therapy and numerous chemotherapy drugs function by inducing tumor cell death through their pro-oxidant effects on DNA. Beta-carotene supplements could potentially disrupt this process, diminishing the effectiveness of the treatment (25) (Table 2).

**Table 2:** Therapeutic controversies over use of beta-carotene supplement during cancer treatment.

Author	Year	Study type	Sample size	Review findings
Margalit, Kasperzyk et al.	2012	RCT	383	During radiation therapy, use of Beta-carotene did not raised risk of prostate cancer, with a 10-year disease-free survival rate of 92% compared to 89% in the placebo group.
Meliante, Petrella et al.	2023	Systematic review	678 participants	Adjuvant therapy using interferon-α, 13-cis-retinoic acid, and α tocopherol showed a 0.90 relative risk of second primary tumors, due to higher lung cancer incidence. No antioxidants were effective in preventing second primary tumors. No significant variations b/n the placebo and 50 mg/day β-carotene receiving groups. In addition, taking 75 mg/day for 3 months has no significant differences in 10-year disease-free survival for locally advanced HNSCC
Story, Sabin, et al.	2019	Guest Post	1 reviewed article	During chemotherapy, use of carotenoids was found to increase the risk of death and recurrence.
Ambrosone, Zirpoli et al.	2020	RCT	1, 134 patients	During chemotherapy, taking carotenoids raises breast cancer recurrence by 41%, while also increasing risk of death.
Nielsen, Kristensen, et al.	2021	Cross-sectional study	1,089 patients	During chemotherapy and radiation treatment, taking antioxidant supplements has been linked to a higher death risk and a lower chance of cancer-free survival in postmenopausal breast cancer survivors.
Cockfield and Schafer	2019	Systematic review of clinical trials	29, 000 patients	Beta carotene consumption increased the incidence of lung cancer by 18% over an eight-year clinical trial.

### 3.3 Therapeutic controversies over use of vitamin C supplement during cancer treatment

Vitamin C supplements are often taken as immune system boosters, especially during cancer treatment (30). Vitamin C supplements use varying formats. Data is conflicted about this, with some studies showing benefits and others potential risks (31).

Among breast cancer patients, there wasn’t an association between use of vitamin C supplements during treatment and chemotherapy-induced peripheral neuropathy (IPN) (19).

In a similar study, people who use vitamin C as an antioxidant had a hazard ratio (HR) of 0.82, with a 95% confidence interval (CI) of 0.65–1.02. This means an 18% lower risk of death and a 22% lower chance of cancer recurrence (HR = 0.78, 95% CI: 0.63–0.95) (32). Another study discovered that using antioxidant vitamin C could lower the risk of breast cancer coming back again (11).

Additionally, taking vitamin C supplements has also been tied to a lesser chance of dying from anything, death due to cancer specifically, or having breast cancer return again (33).

A clinical test also found that combining pharmacological ascorbate with platinum-based chemotherapy helped with tumor shrinkage in patients with advanced non-small cell lung cancer. The objective response rate was 34.2%, and the disease-control rate was 84.2% (18). Similarly, a study showed that adding pharmacological ascorbic acid along with mFOLFOX6 or FOLFIRI lowered gastrointestinal side effects significantly and reduced bone marrow problems in patients of various grades (18).

According to Franziska et al. (34), giving intravenous vitamin C may have shielded against blood-related issues when treating patients with pancreatic cancer rather than using radiation therapy plus temozolomide therapy for glioblastoma alone in one study group’s research results. Another study found that high doses of intravenous vitamin C given to cancer patients displayed better quality of life and four times longer survival periods on average. However, this study did not have a group given a placebo for comparison purposes (35).

Nonetheless, meta-analyses showed no clear difference in general survival or antioxidant use post-chemo treatment [HR1.15; CI: 0.78–1.68; (21)]. Similar studies found that taking vitamin C did not improve the overall survival or health conditions of cancer patients across 19 clinical trials (33, 36).

Similarly, it was suggested in another study that the co-administration of the antioxidant vitamin C with chemotherapy drugs such as cisplatin and doxorubicin could decrease ROS necessary for medication-mediated cell death in more than 10% of subjects (37).

Further to this finding, being involved in trials with vitamin C did not show any significant changes in bio-oxidative indicators or pharmacodynamics endpoints (20). Additionally, contrastive worst endpoints were observed in trial use of anti-oxidants concurrent with chemotherapy (22).

Soluble vitamins such as Vitamin C are especially supportive of resisting the damage wrought upon our non-stop oxidation and inflammation by free radicals (18).

When treating cancer stem cells, vitamin C use impacts energy processes, cancer controls change positively, and intake could work alongside other chemotherapy therapy medications (38).

Reports have suggested that by taking vitamin C supplements, our T-cells can grow when dealing with infections, followed by more cytokine production and antibody creation. The way our body regulates inflammation may be largely influenced by vitamin C (39, 73).

However, taking Vitamin C during chemotherapy might reduce how well the treatment works because it shields out lipid peroxidation linked to chemo drugs like Tamoxifen.

Vitamin C can also cause mitochondrial potential levels to drop within cancer cells (40) (Table 3).

**Table 3:** Therapeutic controversies over use of vitamin C supplement during cancer treatment.

Author	Year	Type	Sample size	Review findings
Subramani, Yeap et al.	2014	Clinical trial	Not mentioned	During cancer treatment, taking vitamin C enhance immunity.
Dębska-Szmich and Potemski	2021	Systematic review	99	Vitamin C intake has been linked to inconsistent findings, i.e., some suggesting benefits and others raising risks.
Zirpoli, McCann, et al.	2017	Prospective cohort	1,225 participants	No association was found between vitamin C intake and chemotherapy-induced peripheral neuropathy.
George and Abrahamse	2020	Systematic review	130 articles	Vitamin C intake decreases risk of death and recurrence among patients with breast cancer by 18 and 22%, respectively.
Chen, Huang, et al.	2022	Umbrella review	22 reviewed articles	Vitamin C intake was linked to decreased risk of breast cancer prognosis, i.e., recurrence, cancer-specific death and all-cause mortality.
Talib, Ahmed Jum’AH et al.	2024	Systematic review	158 articles	Ascorbic acid can improve tumor decline and decrease GI toxic effects and damage of bone marrow in platinum-based chemotherapy.
Franziska, Andrea et al.	2021	Systematic review	57 clinical trials	taking IV vitamin C can protect against hematologic toxicities of radiation and temozolomide therapy during pancreatic cancer treatment.
Cantley and Yun	2020	Systematic review	Not mentioned	IV high-dose vitamin C treatment improved survival time and quality of life by four times without a control group in cancer patients.
Li, Lin, et al.	2021	Systematic review and meta-analysis	8 studies	A meta-analysis showed no significant variations in overall survival or antioxidant supplement intake after chemotherapy (HR 1.15, 95% CI 0.78–1.68).
van Gorkom, Lookermans et al.,	2019	Systematic review and meta-analysis	19 studies	Vitamin C intake did not improve overall survival rate or clinical status.
Chen, Du et al.	2022	A two-sample Mendelian randomization study	10 studies	Vitamin C intake did not improve their overall survival rate or clinical status.
Kranjcec, Abdovic et al.	2022	Cross-sectional study	126 participants	Combining vitamin C with cisplatin and doxorubicin can neutralize ROS needed for cytotoxic effects in over 10% of participants.
Meliante, Petrella et al.	2023	Systematic review	65 articles	Vitamin C have not shown any improvement in oxidation indicators or therapeutic effects in intervention trials.
Story, Sabin et al.	2019	Guest post	1 reviewed article	During chemotherapy, the intake of antioxidants did not result in worsening of endpoints.
Satheesh, Samuel et al.	2020	Systematic review	137 articles	Vitamin C intake effectively impedes with energy metabolism and cancer epigenome control in when combined with another chemotherapeutics.
Carr and Maggini	2017	Systematic review	256 articles	Vitamin C intake have been found to enhance T-cell proliferation secondary to infection, resulting in elevated cytokine production and immunoglobulin production.
Sorice, Guerriero et al.	2014	Mini review	6 articles	Vitamin C intake can significantly affect the control of the inflammatory response.

### 3.4 Therapeutic controversies over use of vitamin D supplement during cancer treatment

Vitamin D can prevent oxidative and cellular damage, which can reduce the detrimental effects of chemotherapy and radiotherapy. For example, high-dose vitamin D supplementation (8,000 IU/day vs. 400 IU/day) enhances survival without deterioration in colorectal cancer patients, decreases diarrhea (grades 3 and 4) and toxic effects, and maximizes the ability to control illness rates, which are 96% in the high-dose group and 84% in the low-dose group (41). The same authors also reported that having normal vitamin D levels was linked to better outcomes for many cancer patients, improved quality of life, and overall health (41).

Among patients with early-stage adenocarcinoma with low levels of vitamin D, a study concluded that vitamin D supplementation significantly outscored the placebo group for five-year survival (86% vs. 50%, *p* = 0.04) and overall survival (91% vs. 48%, *p* = 0.02) (18).

On the other hand, a recent analysis of 16 studies including breast cancer patients found that taking 5 to 25 g of vitamin D daily did not control the chemotherapy-induced loss of bone mineral density (42).

In addition, excessive vitamin D intake can result in hypercalcemia, toxicity, renal damage, and overdose, which can all be fatal. Additionally, these dosages can increase the risk of kidney stones, particularly in those with renal impairments. Likewise, there is a chance that vitamin D will interact negatively with other minerals, including phosphorus, magnesium, and calcium, leading to imbalances (43).

Furthermore, there is a chance that vitamin D will interfere with some chemotherapy medications, reducing their effectiveness or increasing the likelyhood of adverse effects. For instance, vitamin D can intensify the adverse effects of some chemotherapy drugs, such as doxorubicin (44).

Similarly, when vitamin D was used together with other supplements or drugs, high dosages of vitamin D cannot prevent the development of cancer; it can significantly increase cardiovascular disease, risk of osteoporosis, exhaustion, muscle weakness, and painful bones (44).

Vitamin D supplements play significant roles in controlling the tumorigenesis pathway via numerous cellular behaviors such as increased maturation, differentiation, proliferation, and apoptosis, epithelial-mesenchymal transition (EMT), and autophagy along with modulating interactions between cells and their microenvironment (18). However, the use of antioxidant vitamin D supplements may reduce the efficacy of chemotherapy and radiotherapy by decreasing the amount of reactive oxygen species and apoptosis (42) (Table 4).

**Table 4:** Therapeutic controversies over use of vitamin D supplement during cancer treatment.

Author	Year	Study design	Sample size	Review findings
Ng, Nimeiri, et al.	2019	RCT	139 patients	High-dose Vitamin D intake decreased chemotherapy and radiotherapy induced detrimental effects, i.e., diarrhea, and improved survival, maximized disease control and overall health.
Harvie	2014	Systematic review	16 RCTs	Daily vitamin D intake did not inhibit loss of chemotherapy-induced bone mineral density among patients with breast cancer.
Chandler, Chen et al.	2020	Multi-left clinical trial	25,871 patients	Excessive vitamin D intake resulted in hypercalcemia, kidney stones, toxicity, renal damage, overdose due to possible interactions with other minerals.
Scragg, Khaw et al.	2018	Randomized clinical trial	5,108 participants	Vitamin D potentially impedes certain chemotherapeutics, decreasing their efficacy or increasing the risk of detrimental effects.
Talib, Ahmed Jum’AH et al.	2024	Systematic review	158 articles	Vitamin D intake increased 5-year risk-free survival and overall survival in patients with early-stage adenocarcinoma having decreased vitamin D levels compared to a control group.

### 3.5 Therapeutic controversies over use of vitamin E supplement during cancer treatment

The effects of vitamin E supplements on cancer patients have been studied, especially those who are receiving radiation and chemotherapy. Because of its strong antioxidant effect, vitamin E is important for the immune system and endothelial functioning as well as protection of cells and tissues from free radicals by inhibiting fat oxidation (18).

Based on a phase 2 trial including 45 HNSCC patients, a combined regimen consisting of isotretinoin, interferon α-2a, and vitamin E prevents secondary cancers and relapse, which is an effective prevention (20).

Similarly, vitamin E intake has been shown to decrease the likelihood of breast cancer recurrence (11). Likewise, a recent RCT found minimal noninvasive bladder cancer recurrence with vitamin E supplementation (42). Furthermore, a cohort of patients with breast cancer who received vitamin E supplements during treatment had no significant association with chemotherapy-induced peripheral neuropathy (19).

Another study reported that vitamin E supplement users had a decreased risk of death by 18% (hazard ratio (HR) = 0.82, 95% CI: 0.65–1.02) and a lowered risk of recurrence by 22% (HR = 0.78, 95% CI: 0.63–0.95) (32). Furthermore, vitamin E supplementation decreased damage of DNA in breast cancer patients receiving chemotherapy (45).

A prospective, double-blinded, randomized, placebo-controlled study showed that short-term supplementation with vitamin E has a protective effect against radiation therapy-induced dry-mouth (Kim (46),).

Likewise, with the suspension of supplements, the experimental group showed a reduced incidence of secondary neoplasms (39/1000 versus 69/1000, HR = 0.57, 95% CI = 0.31 to 1.0), and it was significantly lower when α-tocopherol was the only supplement (HR = 0.41, 95% CI = 0.16 to 1.03) (20). The same authors examined if the link between smoking and supplements had an effect on secondary tumors and examined the smoking habits of the study participants.

When radiation therapy began, 343 out of 540 patients (60% of the total) smoked; this percentage dropped to 33%. Merely smokers were shown to have a higher frequency of outcomes linked to supplementation: HR 2.41 (95% CI: 1.25–4.64) for recurrence, HR 2.26 (95% CI: 1.25–4.64) for all-cause mortality, and HR 5 3.38 (95% CI: 1.11–10.34) for deaths associated with initial detection of head and neck squamous cell carcinoma (20).

However, meta-analysis study found no apparent disparity in overall survival or the usage of vitamin E supplements during chemotherapy (HR 1.15, 95% CI 0.78–1.68) (21). In addition, a study from a clinical trial that included over 35,500 males over 50 years of age discovered that high vitamin E dosages raised the risk of prostate cancer by 17% (28).

Moreover, a multicentric, double-blind, placebo-controlled, randomized trial that used the same methodology found that in patients who are suffering from stage I or II HNSCC and on radiation therapy while taking 400 IU of α-tocopherol daily had a significantly higher incidence of subsequent primary malignancies (60/1000 person-years) compared to those on placebo (25/1000 person-years); HR = 2.42 (95% CI = 1.45 to 4.04) particularly with only delivery of α-tocopherol (HR = 2.88, 95% CI = 1.56 to5.31) (20). Another study by Donnelly et al. (47), which reviewed RCTs done on alpha-tocopherol usage among head and neck cancer patients showed an increase in cancer recurrence rates.

Another Mendelian Randomization study also indicated that a higher danger of bladder cancer was connected with greater levels of vitamin E in blood (48). In addition, no evidence had been found by Zhang et al. (49) that vitamin E intake is significantly correlated with incidence of breast cancer. Similarly, one antioxidant used for chemotherapy, which is vitamin E did not present any statistically significant relations (22).

Some clinical researches have shown that alpha-tocopherol may decrease the amount of radiation-induced tissue damage while other clinical trials have reported an increased risk of recurrent cancers (50).

Similarly, vitamin E has been demonstrated to reduce radiation damage among patients suffering from head and neck cancer but on the other hand it has been associated with higher risks of recurrence especially among smokers since smoking elevates tissue hypoxia and blood carboxyhemoglobin levels thereby reducing oxygen dependent radiotherapy action (42). Moreover, vitamin e protects against chemotherapy caused injury through inhibiting lipid peroxidation and triggering death of neoplastic cells in tumors (51) (Table 5).

**Table 5:** Therapeutic controversies over use of vitamin E supplement during cancer treatment.

Author	Year	Study design	Sample size	Review findings
Talib, Ahmed Jum’AH, et al.	2024	Systematic review	158 articles	Vitamin E intake prevent free radicals from damaging the cells and tissues during radiation and chemotherapy.
Meliante, Petrella, et al.	2023	Systematic review	Clinical trial (Phase II-45 patients)	Taking 400 IU of α-tocopherol daily prevents secondary cancers and recurrence among smokers diagnosed with HNSCC.
George and Abrahamse	2020	Systematic review	130 articles	Vitamin E intake was found to decrease the chance of recurrence among patients with breast cancer.
Harvie	2014	Systematic review	16 RCTs	Vitamin E intake decreases radiation toxicity in head and neck cancer patients, but increases the risk of recurrence especially in smokers due to the effects of increased oxygen-dependent radiation treatment.
Zirpoli, McCann, et al.	2017	Prospective cohort	1, 225 participants	Vitamin E intake did not affect chemotherapy-induced peripheral neuropathy among breast cancer patients.
Nechuta, Lu et al.	2011	Prospective cohort	4, 877 participants	Vitamin E intake decreased the risk of death by 18% and recurrence by 22%.
Suhail, Bilal, et al.	2012	RCT	40 patients	Vitamin E intake has been found to reduce DNA damage in breast cancer patients who underwent chemotherapy.
Kim, Chung, et al.	2016	RCT	45 patients	Short-term vitamin E intake exerts a protective effect against radiation-induced xerostomia.
Li, Lin, et al.	2021	Systematic review and meta-analyses	8 studies (17, 062 patients)	There is no significant difference in overall survival or the use of antioxidant supplements during chemotherapy.
Cockfield and Schafer	2019	Clinical trial	35,500 males	High vitamin E intakes have been linked to a 17% increase in the risk of prostate cancer.
Donnelly, Appathurai et al.	2022	Systematic review	16 RCTs	Alpha-tocopherol has been linked to a higher likelihood of cancer recurrence among head and neck cancer patients.
Xin, Jiang et al.	2022	Longitudinal cohort study	355,543 participants	A higher risk of bladder cancer was linked to elevated levels of vitamin E in the blood.
Zhang Yi, et al.	2023	Umbrella review	27 articles	No significant association between vitamin E supplements and breast cancer development, and the use of a single antioxidant during chemotherapy did not show significant associations.
Story, Sabin et al.	2019	Guest post	1 reviewed article	No significant association between the use of a single antioxidant, vitamin E, and chemotherapy-related adverse effects.
Integrative	2020	Systematic review	7 articles	Alpha-tocopherol has been linked in few clinical studies to decrease radiation-induced tissue damage, but others suggest an raised risk of cancer recurrence.

### 3.6 Therapeutic controversies over use of coenzyme Q10 supplement during cancer treatment

Coenzyme Q10 is important for cellular growth, energy production, and inhibition oxidative damage. It enhances the synthesis of antioxidants, decreases oxidative stress, and maintains vascular health (52).

A study conducted by Rujkijyanont and Inaba (53) investigated whether CoQ10 could keep the hearts of children with acute lymphoblastic leukemia from damage caused by doxorubicin. The researchers found that Coenzyme Q10 can reduce the drawbacks of doxorubicin on the heart.

According to Khalifa et al. (54), the supplementation of CoQ10 and α-lipoic acid inhibited cisplatin-induced nephrotoxicity. In addition, CoQ10 prevented malignant cells of the colon by enhancing the production of ROS and nitric oxide while reducing the expression of apoptotic genes and decreasing the expression of anti-apoptotic genes (55).

Likewise, daily intake (300 mg) of CoQ10 for 3 months has been shown to enhance the work of antioxidant enzymes and decrease inflammatory markers in hepatocellular carcinoma patients underwent surgery (56).

Meanwhile, it has been reported that supplementing CoQ10 decreased the adverse effects of breast cancer caused by CoQ10 deficiency (57). Furthermore, CoQ10 was not linked with side effects during chemotherapy, and did not worsen either endpoint (22).

In spite of the antioxidant effects of CoQ10 and its use in cancer treatment, there are issues that CoQ10 may decrease the efficacy of these treatments.

The American Cancer Society has warned that CoQ10 counteracts with chemotherapy and radiation therapy, making cancer physicians not to recommend during conventional cancer treatment (58).

The antioxidant CoQ10 can increase the chance of disseminating cancer cells by protecting the malignant cells from oxidative stress, preventing apoptosis, and increasing cancer cell growth. This raises questions on the use of CoQ10 in cancer patients having treatments that depend on the production of free radicals to eliminate cancer cells (59) (Table 6).

**Table 6:** Therapeutic controversies over use of coenzyme Q10 supplement during cancer treatment.

Author	Year	Study design	Sample size	Review findings
Sood, B. et al.	2024	Systematic review	55 articles	CoQ10 is important for energy production, cell growth, and protection against damage of ROS.
Rujkijyanont and Inaba	2024	Systematic review	104 articles	CoQ10 has minimized the adverse effects of doxorubicin on the heart.
Khalifa, Nabil Ahmed, et al.	2020	Clinical trial	40	When CoQ10 combined with ALA, effectively prevented cisplatin-induced nephrotoxicity.
Jang, Lee, et al.	2017	Vitro-study	Not specified	Coenzyme Q10 prevents colon malignant cells by promoting ROS and nitric oxide production, gene expression, controlling apoptotic and decreasing antiapoptotic gene expression.
Liu, Huang et al.	2015	RCT	41 patients	Daily intake of 300 mg of Co Q10 for 3 months has been shown to elevate antioxidant enzyme activity and minimize inflammatory markers in patients with HCC.
Tafazoli	2017	Systematic review	24 articles	CoQ10 intake can minimize the negative effects of breast cancer caused by a CoQ10 deficiency.
Story, Sabin et al.	2019	Guest Post	1 reviewed article	CoQ10 was not linked to adverse effects of chemotherapy and did not worsen either endpoint.
Hu, Huang, et al.	2021	*In vivo* study	Not specified	CoQ10 protected melanoma cells against apoptosis by vemurafenib.

### 3.7 Therapeutic controversies over use of selenium supplement during cancer treatment

Selenium decreases inflammation, prevents DNA damage, controls the production of active thyroid hormone, and regulates a wide range of biological functions, including cell membrane and integrity energy metabolism (13).

In addition, selenium helps to decrease nausea, diarrhea, and lymph retention in extremities that are caused by the adverse effects of cancer treatment (60).

Selenium can reduce the detrimental effects of radiotherapy, as selenoproteins have a function of cytokine regulation, DNA repair, and anti-oxidative characteristics against radiation-induced free radicals (61).

A systematic review showed that daily dosages of 300–500 μg/day of selenium over 10 days to 6 months improved patient conditions and reduced radiation adverse effects. The investigators showed that there were no reported toxicities, and supplementing with selenium did not decrease the efficacy of radiation treatment (61).

A similar study found that selenium supplementation decreased the adverse effects of radiation treatment, improved the patient’s overall health and quality of life. The trials found that supplementing with selenium at the 200–500 μg/day level did not affect the efficiency of radiation treatment, and there were no observed toxicities (62).

In addition, a randomized study of radiation patients with uterine carcinomas found that selenium supplementation effectively reduced radiotherapy-induced diarrhea (63). Furthermore, selenium supplementation did not interfere the effecacy of radiation (63). A second randomized study including patients with head and neck cancer found some beneficial benefits of supplementary selenium in the prevention of swallowing difficulty and loss of taste brought on by radiation therapy (63).

In contrast, an umbrella review found no conclusive evidence that supplementing cancer patients with selenium improves the adverse effects of cancer therapy. The same study revealed that a higher risk of skin malignancies other than melanoma may be linked to selenium use (64).

Another study also reported that selenium supplementation has not been found to prevent prostate cancer as a chemo-preventive agent in randomized controlled trials (HR: 0.95; 95% CI: 0.80–1.13) (65).

Although few studies have reported that selenium can reduce the adverse effects of cancer treatments without affecting their efficacy, they lack consistent findings yet over the type and dosage of selenium for these patients, with different responses seen between normal and malignant cells (66).

The possible reason for the inconsistent findings can be explained by various factors including cancer type, drug dosage, synergism, bioavailability, patient’s lifestyle, tendency to take supplements, and the duration of the studies with other variables incorporated (67) (Table 7).

**Table 7:** Therapeutic controversies over use of selenium supplement during cancer treatment.

Author	Year	Study design	Sample size	Review findings
Rataan, Geary, et al.	2022	Systematic review	72 articles	Selenium is good for human health, protecting cells and decreasing detrimental effects of cancer treatment, i.e., diarrhea, lymph retention in limbs, and nausea.
Handa, Puspitasari et al.	2020	Systematic review	8 articles	Selenium can minimize radiotherapy adverse effects by repairing DNA, possessing anti-oxidative properties, regulating cytokines, and improving nutritional conditions of patients and decreasing radiation adverse effects with daily doses.
Yasueda, Urushima et al.	2016	Systematic review	49 articles	Selenium intake reduces inflammation, regulates thyroid hormone synthesis, protects against DNA damage and affects energy metabolism and membrane integrity.
Puspitasari, Abdulah et al.	2014	Systematic review	16 articles	Selenium intake decreased radiation treatment detrimental effects, improved quality of life and did not affect treatment efficacy or cause toxicities.
Muecke, Micke, et al.	2018	Systematic review	2 randomized phase III clinical studies	Selenium intake decreases radiotherapy-induced diarrhea in patients with uterine carcinoma, without affecting radiation efficacy. Additionally, it prevented difficulty in swallowing and loss of taste in patients with head and neck cancer.
Wang, Chen, et al.	2023	Umbrella review	76 meta-analyses	No conclusive evidence was found that selenium intake improves adverse effects of cancer therapies, but may increase the risk of skin malignancies other than melanoma.
Jiang, Chen, et al.	2023	Systematic review	7 RCTs	As a chemo-preventive agent, selenium intake has not been proven to prevent prostate cancer.

### 3.8 Therapeutic controversies over use of zinc supplement during cancer treatment

Zinc has anti-oxidative characteristics that lead to a reduction of free radicals and improved human health (68).

There have been scientific reports that supplementation of zinc decreases mucositis, i.e., decreased dry mouth, minimal oral pain, reduced loss of taste caused by radiation toxicities (69).

In addition, supplementation of zinc decreases oxidative stress biomarkers and inflammatory cytokines by preventing protein, DNA and RNA oxidation, as well as blocking inflammatory responses (68, 70).

Furthermore, unregulated status of zinc leads to oxidative stress, cell cycle progression, damage of DNA, and angiogenesis, finally resulting in hepatocarcinogenesis (71). Similarly, the findings of a systematic review found no significant side effects linked to zinc supplementation (13).

To the contrary, zinc supplementation did not alleviate mucositis or improve quality of life or survival rates after chemotherapy (69).

A systematic review also showed that supplementation of zinc did not significantly decrease symptoms of radiotoxicity such as weight loss, changes in taste, nausea, and vomiting (13).

It is important to bear in mind that the efficacy of zinc supplementation depends on factors like individual study characteristics, publication bias, sample size, interactions with other nutrients, differences in methodology, and timing of supplementation. This difference could contribute to inconsistent outcomes (72) (Table 8).

**Table 8:** Therapeutic controversies over use of zinc supplement during cancer treatment.

Author	Year	Study design	Sample size	Review findings
Prasad and Bao	2019	Systematic review	191 articles	Zinc intake was found as anti-oxidative stress agent and reduce ROS production, inflammatory responses by promoting human health.
Hoppe, Kutschan et al.	2021	Systematic review	19 articles	Zinc intake decreases symptoms of mucositis, i.e., dry mouth, taste loss and oral pain.
Prasad	2014	Systematic review	32 articles	Zinc intake decreased oxidative stress biomarkers and inflammatory cytokines by inhibiting protein, DNA, RNA oxidation and as well as decreasing inflammatory responses.
Himoto and Masaki	2024	Systematic review	188 articles	Impairment of zinc can lead to cell cycle progression, oxidative damage, angiogenesis, DNA damage, and hepatocarcinogenesis.
Yasueda, Urushima et al.	2016	Systematic review	49	Zinc intake did not decrease adverse effects, i.e., weight loss, taste alteration, nausea, and vomiting, nor alleviate radiotoxicity.

## 4 Conclusion and recommendations

### 4.1 Conclusion

According to this review, the use of antioxidant supplements can benefit tumor cells in the same manner as they do for normal cells. In addition, how chemotherapeutic drugs and radiation destroy cancer cells varies greatly, with distinct adverse effects, being stressful to different organs, and being toxic to humans over longer periods. Furthermore, not all antioxidants are believed to be effective. Therefore, as only a few clinical studies with small sample size, limited populations, cancer types, stages, dosages etc. have demonstrated the use of antioxidant supplements in cancer treatment, physicians should advise their patients not to take antioxidant supplements during chemotherapy or radiotherapy.

### 4.2 Recommendations

Based on the review findings, it is recommended that physicians need to stay up-to-date on the latest research in this fast-moving field of research so they can advise their patients about potential benefits and risks. Likewise, patients should receive personalized supplemental advice from a credible source given by their cancer physician. In addition, future research including potential clinical and preclinical trials, mechanistic studies, and exploration of different vitamin and mineral supplement studies are required to uncover the complete potential of antioxidant supplements for cancer treatment or determine their safety and effectiveness when used alongside standard cancer treatments. Furthermore, the results of this review could be used for future systematic review of therapeutic controversies over use of antioxidant supplements during cancer treatment.

### 4.3 Strengths and limitations

Strengths of the present review include an extremely rigorous search strategy intended to capture the full range of publications presenting data on this topic. *A priori* inclusion criteria and duplicate screening decreased the risk of bias. The very large scope of this review was both a strength and limitation. Due to the very large volume of articles included in the review, in-depth analysis of individual articles was not possible. In addition, the reviewer gathered English-language studies on antioxidant supplements’ therapeutic controversies during cancer treatment, primarily from North and South America, Europe, and Asia, without considering cultural and socioeconomic disparities, requiring caution in application.

Another limitation that impacts the ability to draw clear conclusions from the present data is the enormous complexity of studying all the available antioxidant supplements in medical and nutritional science.

## Glossary

AHR: Adjusted hazard ratio
ALA: Alpha-lipoic acid
β-hCG: Beta-human chorionic gonadotropins
CAT: Catalase
CI: Confidence interval
CIPN: Chemotherapy induced peripheral neuropathy
CRC: Colorectal cancer
CoQ10: Coenzyme quotient 10
DNA: Deoxyribose-nucleic acid
EMT: Epithelial-mesenchymal transition
GPx: Glutathione peroxidase
HNSCC: Head and neck squamous cell carcinoma
HR: Hazard ratio
IVC: Intra venous vitamin C
IU: International unit
MTD: Maximum tolerated dose
NSCLC: Non-small cell lung cancer
PSA: Prostate surface antigen
QoL: Quality of life
RCT: Randomized controlled trials
ROS: Reactive oxygen species
RP2D: Recommended phase 2 dose
RR: Relative risk
RT: Radiation therapy
SOD: Superoxide dismutase
TMZ: Temozolomide
WHO: World Health Organization
